# Supplementary material for: Transcriptional Regulation of Liver-Type OATP1B3 (Lt-OATP1B3) and Cancer-Type OATP1B3 (Ct-OATP1B3) Studied in Hepatocyte-Derived and Colon Cancer-Derived Cell Lines
Source: Pharmaceutics. 2023 Feb 23;15(3):738. doi: 10.3390/pharmaceutics15030738 (PMC10051083; doi:10.3390/pharmaceutics15030738)
Supplement: Supplementary file 1 [file pharmaceutics-15-00738-s001.zip › pharmaceutics-2145899-supplementary.pdf]

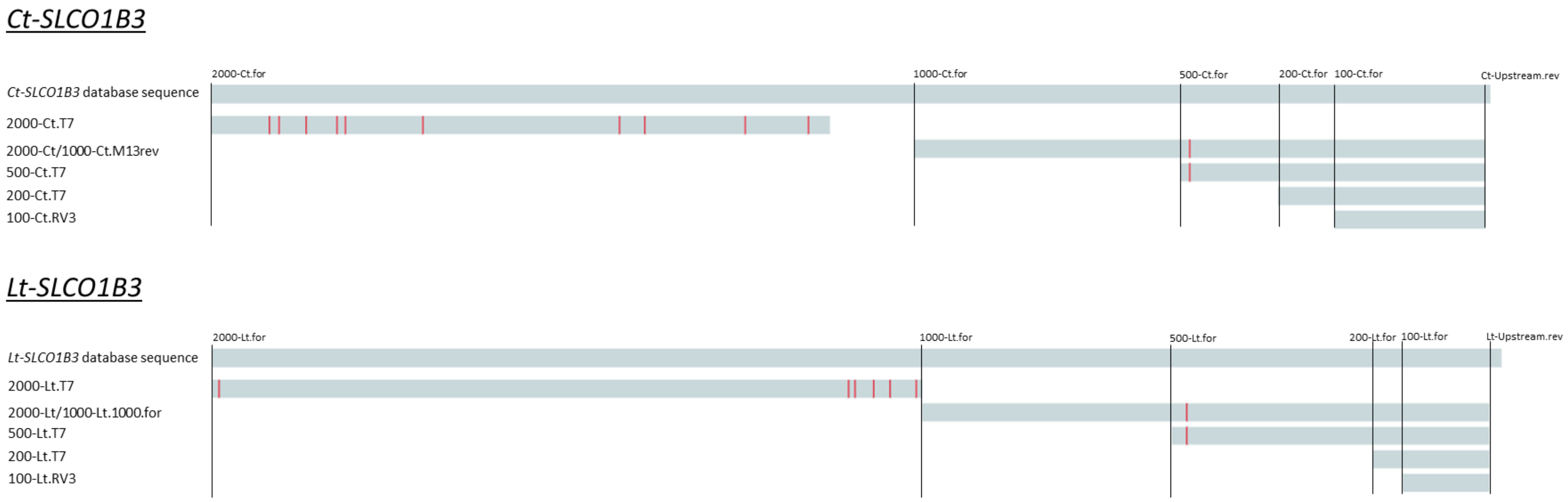

Figure S1: DNA sequence analysis

The light blue areas indicate sequence identity with the respective database sequence, red lines show localization of single base pair differences. DNA alignment was performed using the benchling.com web page (https://benchling.com/). The sequences used for the alignment are listed below.

Ct-SLCO1B3 database sequence:

gtggctggggtttgtctcacagtggaggcaaggaattgcaactttgttttattactgtacaccttgaaagtgaggttaattaagtcctgttatggggt
ttgagggccagattccaatttttggagttttatttaatgtcaggagcagattgggtaataaaatgtatattgagaataagatggccttttgaccttttt
agggtctagggctgtaaagcatctcagggttgctgccgaatgagccatgaactgggctgggtttttatatttgatgaaaaagagcctaaacgcttc
tgatttgggataaagaaaaaggagcattaaccttgactatgcctttggctccagccacctttttaagagtaaattgctgggcaggtgggggaggg
gtagtcatggaacaaaactgtaagccggatcaggtgtgaggaggggaggcgataaaaagtttatagggtggaggagcagaggctgaggaaga
attgggaactagctcggcctggcgaggaggggagaggtcagatgggtctgtagaaaaggaagattagaaagactcagcgacgcttggggttgg
gcctgaggggacaggcgggagggaaagaaggaagatttgggatgaattgcattgggcagagactaggaagggactgatgtgtaaaagaatgc
ctggacatcaggcacctcagaccatttgcccgttttacgacaagaattatttaggtcttgtaggatggaaaaattgaaagtgccgtttttctggcaa
tttgtaactactgtcgagtttgtactggggtcaagcggcattgcagaagaaaaaagacgcttagattttaggtcaggtgagagttgaagaggtttt
aagttcttaagaatacaggctaagggagaagaaggaggaatggaaggtggaagctagtccatagtgaaggaggcaagcccagagaaaagag
tagagacacggagaagtgatgggggtttcttgccctccagaaaagcagagaaagggttggggcacggaaataagggattgggggttcttgccc
cctagaaaagcgggacttgccactaagggtgaaggagaaggggttgaggggtacttgcccctcccccagaaaagcagagaaggggtagagac
atggagagaaggggttggggtacttgcccctcccccagaaaagcgggacttgcggctaagggtgaaggaccaaggcaggcatccctgcgtggt
ctgacacctttgaaatgtgggtgaataatcagagaggcatccctgcaatgattaaacaccaagggaaggctgccttcccagtccgtgaccggcac
cggagttttgggtccacggataaaacatgtctcctttgtctctatccgaaaatgaaaggaattgaaattaagagaagggagagattgaagtgtgg
tgtcaagattgaaaggagaaagaggttgagggatagtgaaggaagttggagaagagagtaaaaagaggccgcttaccggatttgaaattggtg
agatgtttcttgggctggtcggtctgaggacctgaggtcgtagatggatctttctcacggagcaaagagcaggaggacgggggattgatctccca
agggaggtcccccgatccaagtcacggcaccaaatttcatgtgtgtccatgtgaagagaccaccaaacaggctttgtgtgagcaataaagctgtt
tatttcacctggatgcggttgggctgagtccgaaaagagagtcagtgaagggagataagggtggggccgttttataggatttgggaaggtaaag
gaaaattacagtcaaagggggttgttctctggtgggcaggggcgggggtcacaaggtgctcggtgggggagcttctgagccaggagaaggaaa
ttcaaagggttagtcactcagttaaggtggggcaggaacaaatcacaatggtggaatgtcatcagttaaggcggggcagggcctttcacttctttt
gtgattcttcagttacttcaggccatctcggcgtatacgtgcaagtcacaggggatgggatggcttggcttgggctcagagacctgacagtggcaa
tgtatggccacgttactgaatctacatgttgcaagagaaaaactagcaggtaagggaatggtcagttatttatttg

2000-Ct.T7

ggcggccctgatgcatgctcgagcggccgccagtgtgatggatatctgcagaattcgccctttggaggcaaggaattgcaactttgttttattactg
tacaccttgaaagtgaggttaattaagtcctgttatggggtttgagggccagattccaatttttggggttttatttaatgtcgggagcagattgggta
ataaaatgtatattgagaataagatggcctttgaccttttagggtctagggctgtaaagcatctcagggttgctgccgagtgagccatgaactggc
tgggtttttatatttgatgaaaaagagcctaaacgcttctgatttgggataaagaaaaaggagcattaaccttgactatgcctttggctccagccac
ctttttaagagtaaattgctgggcaggttggggaggggtagtcatggaacaaaactgtaagccggatcaggtgtgaggaggggaggcgataaa
aagtttatagggtggaggagcagaggctgaggaagaattgggaactagctcggcctggcgaggaggggagaggtcagatgggtctgtagaaa
aggaagattagaaagactcagcgacgcttggggttgggcctgaggggacaggcgggagggaaagaaggaagatttgggatgaattgcattgg
gcagagactaggaagggactgatgtgtaaaagaatgcctggacatcaggcacctcagaccatttgcccgttttacgacgagaattatttaggtct
tgtaggatggaaaaattgaaagtgctgtttttctggcaatttgtaactactgtcgagtttgtactggggtcaagcggcattgcagaagaaaaaaga
cgcttagattttaggtcaggtgagagttgaagaggttttaagttcttaagaatacaggctaagggagaagaaggaggaatggaaggtggaagct
agcccatagtgaaggaggcaagcccagagaaaagagtagagacacggagaagtgatgggggtttcttgccctccagaaaagcagagaaagg
gttggggcacggaaatgagggattgggggttcttgccccctagaaaagcggga

2000-Ct/1000-Ct.M13rev

taactgaccattcccttacctgctagtttttctcttgcaacatgtagattcagtaacgtggccatacattgccactgtcaggtctctgagcccaagcc
aagccatcccatcccctgtgacttgcacgtatacgccgagatggcctgaagtaactgaagaatcacaaaagaagtgaaaggccctgccccgcct
taactgatgacattccaccattgtgatttgttcctgccccaccttaactgagtgactaaccctttgaatttccttctcctggctcagaagctcccccac
cgagcaccttgtgacccccgcccctgcccaccagagaacaaccccctttgactgtaattttcctttaccttcccaaatcctataaaacggccccacc
cttatctcccttcactgactctcttttcggactcagcccaaccgcatccaggtgaaataaacagctttattgctcacacaaagcctgtttggtggtctc
ttcacgtggacacacatgaaatttggtgccgtgacttggatcgggggacctcccttgggagatcaatcccccgtcctcctgctctttgctccgtgag
aaagatccatctacgacctcaggtcctcagaccgaccagcccaagaaacatctcaccaatttcaaatccggtaagcggcctctttttactctcttct
ccaacttccttcactatccctcaacctctttctcctttcaatcttgacaccacacttcaatctctcccttctcttaatttcaattcctttcattttcggata

gagacaaaggagacatgttttatccgtggacccaaaactccggtgccggtcacggactgggaaggcagccttcccttggtgtttaatcattgcag
ggatgcctctctgattattcacccacatttcaaaggtgtcagaccacgcagggatgcctgccttggtccttc

500-Ct.T7

ttcatgtgtgtccacgtgaagagaccaccaaacaggctttgtgtgagcaataaagctgtttatttcacctggatgcggttgggctgagtccgaaaa
gagagtcagtgaagggagataagggtggggccgttttataggatttgggaaggtaaaggaaaattacagtcaaaggggggttgttctctggtggg
caggggcgggggtcacaaggtgctcggtgggggagcttctgagccaggagaaggaaattcaaagggttagtcactcagttaaggtggggcagg
aacaaatcacaatggtggaatgtcatcagttaaggcggggcagggcctttcacttcttttgtgattcttcagttacttcaggccatctcggcgtatac
gtgcaagtcacaggggatgggatggcttggcttgggctcagagacctgacagtggcaatgtatggccacgttactgaatctacatgttgcaagag
aaaaactagcaggtaagggaatggtcagtta

200-Ct.T7

cagtcaaaggggggttgttctctggtgggcaggggcgggggtcacaaggtgctcggtgggggagcttctgagccaggagaaggaaattcaaagg
gttagtcactcagttaaggtggggcaggaacaaatcacaatggtggaatgtcatcagttaaggcggggcagggcctttcacttcttttgtgattctt
cagttacttcaggccatctcggcgtatacgtgcaagtcacaggggatgggatggcttggcttgggctcagagacctgacagtggcaatgtatggc
cacgttactgaatctacatgttgcaagagaaaaactagcaggtaagggaatggtcagtta

100-Ct.RV3

taactgaccattcccttacctgctagtttttctcttgcaacatgtagattcagtaacgtggccatacattgccactgtcaggtctctgagcccaagcc
aagccatcccatcccctgtgacttgcacgtatacgccgagatggcctgaagtaactgaagaatcacaaaagaagtgaaaggccctgccccgcct
taactgatgacattccaccattgtgatttgttcctgccccaccttaactgagtgactaa

Lt-SLCO1B3 database sequence

acttaggaaatttatactgctgtcaagtagcagagacattggtatcaaagtttatcaaacttcaaagcccataatatttcatcagaactgtgatgat
tattgcggcaacaaacagcagattgttttaagcaaatttggagttgtattgatccacagctgttagcataatatttatcttgaaaaaaagtgttgca
atgtgtttcctctgaccactcttatttttccacaagtcccacaaaaagggaagtctaattacttacataggaagaaaaaagggcacagagaatgc
tctttgacttctgaaaacattccagaattttaccactgatagcgttagtttagggattagaaagtttctcagtgcatttgtactattttatcttcattct
atgcatgtatttgtcccaaatgaaattgatgtgtcttgaatgaaatggatgtgtgaggagaaaaccattagcatgagaatatccaaactgtcttcc
cattctgaataaaagaaaagccataaaactgataattaatgtttagtatctctgctatgatggttctcaaaagctgacagtatgtaaaaattattct
aagtgcttatttaaaaagcataatctccgaactcgtgaatcagaatctcttgggtgtggggctggatcgcacttttgacaaaggctccccagtaga
ttttgactcaacagaggggtgtttgggagcctatatctaattttccctgccccaggtctacacttcagtgttccccacattttctaatccttctctccct
ccacactccggttcctttcattattctaatacttccatgaattaaccaggtatagaactaatctaggctgggcgcagtggctcacgcctgtaatccta
gcactttgggaggccgaggtgggcagatcacgaggtcaggagatcgagaccatcctggctaacatggtgaaaccccgtctctactaaaaatacg
aaaaaattagccgggcgtggtggcgggcgcttgtagtgccagctactcgggagactgaggcaggagaatggcgtgaacccgggaggcggagct
tgcagtgagccgagatcgtgccgctgcactccagcctgggtgacagagcaagactccgtctcaaaaaaaaaaaaaaaaaactagtttatcaaata
actgtgatatagaaattaaatacatattgctgcaactgtatcaacaactgtcctgtcagtgataaggggtaacaatgttttcatacatgcattctctc
tttgctatatacatacacacatacacataaatacatatatacatacactcatgcatgtgtgtgtatatatacatgtgagagaagactccgtttattat
ttatatttttatagtacacttctaaaacacagccatgtgcctgagatatagtagggagtcaaatatgtttgagttactgaattaattaatttgacttca
tccttactcaagaaaagtttcaactgagtgaaggtcttccattctctaaagagcaagtcaatagaaggagatcattcttcctttacacattgatgta
aggaatatatcatacaaactagtcttgatttttgtcatttagaagactagtcttgtaattaattggcaatttagtattccatgtgagatatccagtgtc
catgtacgtcatattagatccatacacatgtaacaagcaaagagggtatgttttatttttgaatgctaatgtaacatgtagggacattttaaaaaa
tcgccttgacatattttctatttggaatagttttaaatactttggataatgtgttgtatttgtatttctaaagaagaaaatcttatgcaaccaacttaat
tgaataagaaataaatcattcccagaaataatttattttctaatgttaacataatgtgtacattctgagaaattattaataaaatgtttaaaagata
ggcttctggggtgaactcctagaattagctaggtatgtttttttctgtttgcctaggacaatgacctaataagatggttaatcatcattggactcataa
aaacaaacaaataaaaagccaactaaccatttaaagtgagactttaacatcagaaaaaggatggacttgttgcagttgctgtagcattcaaagt
caag

2000-Lt.T7

ggccgccttgatgcatgctcgagcggccgccagtgtgatggatatctgcagaattcgcccttggagttgtattgattcacagctgttagcataatat
ttatcttgaaaaaaagtgttgcaatgtgtttcctctgaccactcttattttttccacaagtcccacaaaaagggaagtctaattacttacataggaag
aaaaaagggcacagagaatgctctttgacttctgaaaacattccagaattttaccactgatagcgttagtttagggattagaaagtttctcagtgc
atttgtactattttatcttcattctatgcatgtatttgtcccaaatgaaattgatgtgtcttgaatgaaatggatgtgtgaggagaaaaccattagcat
gagaatatccaaactgtcttcccattctgaataaaagaaaagccataaaactgataattaatgtttagtatctctgctatgatggttctcaaaagct
gacagtatgtaaaaattattctaagtgcttatttaaaaagcataatctccgaactcgtgaatcagaatctcttgggtgtggggctggatcgcacttt
tgacaaaggctccccagtagattttgactcaacagaggggtgtttgggagcctatatctaattttccctgccccaggtctacacttcagtgttcccca
cattttctaatccttctctccctccacactccggttcctttcattattctaatacttccatgaattaaccaggtatagaactaatctaggctgggcgca
gtggctcacgcctgtaatcctagcactttgggaggccgaggtgggcagatcacgaggtcaggagatcgagaccatcctggctaacatggtgaaa
ccccgtctctactaaaaatacgaaaaaattagccgggcgtggtggcgggcgcttgtagtgccagctactcgggagactgaggcaggagaatggc
gtgaacccgggaggcggagcttgcagtgagccgagatcgtgccgctgcactccagcctgggtgacagagcaagactccgtctcaaaaaaaaac
aaaaaactagtttatcaaataactgtgaatagaaattaaatacatattgctgtaactgtatcaacaactgtcctgtcagtgataaggggtaacatg
ttttca

2000-Lt/1000-Lt.1000.for

tacatgcattctctctttgctatatacatacacacatacacataaatacatatatacatacactcatgcatgtgtgtgtatatatacatgtgagagaa
gactccgtttattatttatatttttatagtacacttctaaaacacagccatgtgcctgagatatagtagggagtcaaatatgtttgagttactgaatta
attaatttgacttcatccttactcaagaaaagtttcaactgagtgaaggtcttccattctctaaagagcaagtcaatagaaggagatcattcttcctt
tacacattgatgtaaggaatatatcatacaaactagtcttgatttttgtcatttagaagactagtcttgtaattaattggcaatttagtattccatgtg
agatatccagtgtccctgtacgtcatattagatccatacacatgtaacaagcaaagagggtatgttttatttttgaatgctaatgtaacatgtagg
gacattttaaaaaatcgccttgacatattttctatttggaatagttttaaatactttggataatgtgttgtatttgtatttctaaagaagaaaatcttat
gcaaccaacttaattgaataagaaataaatcattcccagaaataatttattttctaatgttaacataatgtgtacattctgagaaattattaataaa
atgtttaaaagataggcttctggggtgaactcctagaattagctaggtatgtttttctgtttgcctaggacaatgacctaataagatggttaatcat
cattggactcataaaaacaaacaaataaaaagccaactaaccatttaaagtgagactttaacatcagaaaaaggatggacttgttgcagttgc

500-Lt.T7

gcaactgcaacaagtccatcctttttctgatgttaaagtctcactttaaatggttagttggctttttatttgtttgtttttatgagtccaatgatgattaa
ccatcttattaggtcattgtcctaggcaaacagaaaaaacatacctagctaattctaggagttcaccccagaagcctatcttttaaacattttatta
ataatttctcagaatgtacacattatgttaacattagaaaataaattatttctgggaatgatttatttcttattcaattaagttggttgcataagatttt
cttctttagaaatacaaatacaacacattatccaaagtatttaaaactattccaaatagaaaatatgtcaaggcgatttttttaaaatgtccctacat
gttacattagcattcaaaaaataaaacataccctctttgcttgttacatgtgtatggatctaatatgacgtacagggacactggatatctcacatgg

200-Lt.T7

gcaactgcaacaagtccatcctttttctgatgttaaagtctcactttaaatggttagttggctttttatttgtttgtttttatgagtccaatgatgattaa
ccatcttattaggtcattgtcctaggcaaacagaaaaaacatacctagctaattctaggagttcaccccagaagcctatc

100-Lt.RV3

ctgtttgcctaggacaatgacctaataagatggttaatcatcattggactcataaaaacaaacaaataaaaagccaactaaccatttaaagtgag
actttaacatcagaaaaaggatggacttgttgcagttgc
